# The Anatomic Occlusion of Artificial Teeth

**Published:** 1910-03-15

**Authors:** J. H. Prothero

**Affiliations:** Chicago, Ills.


### THE ANATOMIC OCCLUSION OF ARTIFICIAL TEETH.

BY J. H. PROTHERO, D.D.S., CHICAGO, ILLS.

By anatomic occlusion of artificial teeth is meant the selection of teeth of proper size, form and shade to meet the temperamental requirements of the patient, and their arrangement and occlusion in accordance with nature's plan, so that normal jaw movements may be carried out in the mastication of food.

The development of this system, which was conceived before Bonwill's time, has progressed very slowly because of the meager and imperfect understanding of the mandibular movements involved in mastication. The text-books of today contain incomplete and inaccurate descriptions of the jaw movements that are of interest to the prosthetist. Bonwill's efforts and research work, while extending through many years and in many respects of great value, failed to bring out the most vital details. He believed he had solved the problem of anatomic occlusion, and up to the time of his death failed to recognize, or at least admit that there were any imperfections in his system or appliances.

The first really scientific investigation of record relative to jaw movements in the living subject, was conducted by Bowdich and Luce of Harvard in 1889, but owing to the fact that their report appeared only in the Boston Medical and Surgical Journal, it failed to reach or benefit the dental profession. In 1894-5 Dr. W. E. Walker of New Orleans, without knowing what had been accomplished by the investigators mentioned, carried out a similar line of experiments on the living subject. His report was published in the *Dental Cosmos* in 1896-7 and today stands as the best and most complete scientific treatise on jaw movements extant. So far as the records show, Dr. Walker was evidently the first to recognize the importance of measuring the inclination of the condyle path and of the need of an occluding frame capable of rproducing the mandibular movements peculiar to each individual patient.

To occlude teeth anatomically certain conditions must be considered, and a number of important steps carried out. First—It is necessary to understand the mechanism of the masticatory apparatus and of such jaw movements as are normally carried on in the mastication of food. Second—An occluding frame capable of reproducing such movements in a fairly accurate degree is indispensable. Third—The relation of the natural alveolar planes to each condyle must be correctly registered, and the models mounted on the occluding frame so that their alveolar planes bear a corresponding relation to the frame hinges. Fourth—The inclination of the paths followed by the condyles in lateral and protrusive movements must be registered, and the slots on the frame representing these paths must be set at a corresponding angle. Fifth—Judicious and careful selection of teeth to meet the temperamental requirements of each individual case must be made, as previously stated. Sixth—The teeth must be attached to rigid base-plates for trial in the mouth, modified by grinding to correct disproportion in size and form, and to increase occlusal areas as well as to develop shearing properties and triturating power, and all so arranged that the lower teeth may be carried either laterally or forward along the lines of normal jaw movements while contact at several widely divergent points is possible without cusp interference.

In occluding teeth anatomically, three jaw movements must be considered: First—The hinge movement in which the jaw is opened and closed, the condyles remaining in the glenoid cavities and acting as centers of rotation. Second—The lateral movement in which one condyle serves as a pivotal point, the other being drawn forward in its path Third—The protrusive movement in which both condyles move forward along their respective paths toward the anterior margin of the glenoid cavities.

Previous to the investigations of Bowdich and Walker, the idea prevailed that in the protrusive and lateral movements, the condyles moved horizontally forward. Thier investigations clearly proved that the condyle path in almost every instance inclines downward as well as forward, the degree of inclination varying from horizontal to an angle of 50 degrees, the average being about 35 degrees.

Frequently in the same individual there is a marked difference in the inclination of the two paths. Walker cites a case in which there was a variation of ten degrees in the condyle paths of the same individual.

In almost all cases of normal occlusion the tips of the buccal cusps of the bicuspids and molars when viewed bucally, assume a curved arrangement, the convexity being downward. This curve, if projected backward, will very frequently coincide with the curve of the condyle path. In other words the two curves will represent arcs of circles having equal radii and a common center.

When the curved arrangement of the teeth is less marked, it is found that the curve of the condyle path represents the arc of a smaller or inscribed circle having a common center with the arc intersecting the teeth, and hence running parallel with the latter. The curve intersecting the tips of the cusps of the teeth is known as the compensating curve, or the curve "of Spee" from the man who first described it.

The center of these imaginary arcs lies in the region of, or above, the orbit. The flatter the curve, the farther above the orbit will be the center. As the inclination of the condyle path decreases, the curve merges from the arc of a circle into a straight plane. Therefore when the condyle path is horizontal, the plane of occlusin will be flat. The two, while not in the same plane, will be parallel, however.

When the bicuspids and molars are viewed anteriorly, their buccal arrangement being curved as described, it will be found that the lingual cusps of the lower teeth are placed on a lower plane than the buccal cusps, while the lingual cusps of the upper teeth also assume a relatively lower position than the buccal cusps.

Now if an arc be struck from above having the same radius as that of the buccal or compensating curve, and from a center located on the perpendicular median line of the face, it will intersect the tips of the buccal and lingual cusps of the teeth on both sides of the jaw. Again when a condyle path and plane of coclusion from before backwards is horizontal, the lingual and buccal cusps of the teeth in both arches are practically on the same horizontal plane.

Since in the lateral and protrusive movements of the jaw the lingual as well as the buccal cusps and marginal ridges of the lower are brought in contact with the corresponding surfaces of the upper teeth at many points, regardless of the fact that the plane of occlusion may be curved or flat, we are forced to the conclusion that the occlusal surfaces of the upper and lower teeth represent either plane sections of convex and concave spheres respectively, or of two true planes.

In the study of surfaces and solids, it is found that only two forms of surfaces can be made to move freely over each other in any direction without contact being disturbed, viz., a convex against a concave sphere and one plane surface against another. The form and movements of the mandible together with nature's normal arrangement of the teeth, prove conclusively that in the arrangement of artificial teeth one or the other of these planes must be followed. The condyle path, representing as it does the section of an arc struck from the common center of the various curves mentioned, furnishes a true anatomical key to occlusion.

While the protrusive and hinge-like movements of the mandible are useful in certain steps in mastication, the lateral movement is by far the most effective for tearing asunder, grinding and triturating nearly all varieties of food.

In the lateral movement of the jaw one condyle becomes the pivotal point, while the other is projected forward usually about 3 or 4 mm. A forward movement to this extent is sufficient to carry the lower teeth on the opposite or pivotal side, outward far enough to bring their buccal cusps and marginal ridges directly under and in alignment with the corresponding cusps and surfaces of the upper teeth, the lower lingual cusps and marginal ridges sustaining a similar relation to those of the upper teeth also. The extent of this side movement has been very appropriately termed "The Differential" by Dr. T. W. Pritchett.

When contact is effected by the closure of the jaw, the teeth being in the relation mentioned, it will be found that a long rectangular groove extending from the last molar to the first bicuspid is formed by the centrally inclined occlusal planes of the various cusps on the pivotal or working side. The buccal muscles acting externally and the tongue lingually, direct the food into this groove as closure occurs, and as the mandible is drawn back to normal position the food is crushed and torn asunder and under repeated muscular action is insalivated and reduced to a suitable condition for introduction into the stomach.

On the opposite or projected side, the lower teeth are carried lingually until their buccal cusps and marginal ridges are opposite the lingual cusps and marginal ridges of the upper teeth. Contact at this side of the mouth is usually broken except at the extreme distal termination of the two arches.

When the third molars are in position it will be found that the disto-buccal cusp of the lower third molar, occupying a higher position in the compensating curve, is brought forward in contact with the mesio-lingual cusp of the upper third molar which occupies a lower position in the curve mentioned. This is known as the "balancing contact." Without such contact it would be impossible for the full effective force of the muscles of mastication to be exerted in crushing the food on the opposite side of the mouth because of the tipping of the mandible.

The statement has frequently been made, and is still believed by many, that on account of the loose-jointed articulation of the condyles with their respective fossae, and of the great latitude of movement of the mandible in various directions, mastication is accomplished without well defined or systematized jaw movements, the teeth striking those in the opposite arch only occasionally in the same place.

Nothing could be more misleading than this conception of nature's most beautiful and perfect masticatory apparatus. The occlusal surfaces of the teeth present a series of grooves and ridges formed by the cusps and their various sloping planes. These surface markings are so located that they run with almost mathematical precision, in the direction of normal mandibular movements.

The ridges of one denture glide back and forth through the grooves of the opposite to the full extent of the differential, much the same as the cross head of a piston rod moves back and forth in its guides.

In fact instead of the jaw movements determining the position of contact of the teeth in mastication, these alternate ridges and depressions control and guide the jaw movements in effective masticatory effort.

The theoretical phase of this subject has now been covered, briefly it is true, but sufficiently well to present the practical application of these principles to denture construction.

The best method of presentation will be to illustrate the successive steps in the construction of a full upper and lower denture. The models having been secured, metal or other rigid unyielding baseplates are adapted to them. On these baseplates rims of wax are formed representing approximately the length of the teeth and the general contour outline required. These rims should be about 8 or 10 mm. wide across the occlusal and incisal surfaces, and flat from before backward as well as from side to side.

They are now introduced into the mouth and the rims reduced or lengthened, as conditions require, to establish the correct distance between the two borders and accomplish facial restoration. The labial and buccal surfaces are modified to also bring out proper facial contour. The upper rim of wax shonld usually extend about 1½ mm. below the upper lip when the latter is at rest. Variations from this rule, however, are occasionally necessary.

The lower rim should be of such height that when in contact with the opposite rim, the lower lip may rest normally against the upper without requiring any muscular effort to bring it in that position. If the rim is too short the lower lip will be pursed out and depressed; if too long, muscular effort will be necessary to bring it in contact with the upper lip.

Securing the correct length of each wax rim and of normal facial coniour by the development of the labial and buccal surfaces at this time, are points of vital importance, any one of which if neglected or imperfectly carried out, will require much effort to subsequently correct. These various surfaces serve as guides in the incisal, labial and buccal alignment of the teeth, and if incorrect will necessitate changing the position of the latter when once occluded.

The median line of the mouth should be marked on the upper baseplate, sometimes a difficult point to determine but by placing a cardboard or straight edge along the median line of the face and striking an average midway belween the inner ends of the eyebrows, the philtrum of the lip and the point of the chin, the result will usually be harmonious and in keeping with the general features of the face.

The high lip line is secured by having the patient smile broadly, and marking the curvature and height to which the lip is raised on the wax rim. The low lip line is marked in the same manner. These lines serve as guides in selecting teeth of such length that neither too much porcelain or artificial gum will be exposed in laughing. When these steps have all been carried out, the general facial contour should again be reviewed and any corrections made that may be necessary.

The upper baseplate should now be removed, the crescent shaped plate of the stem of the face bow heated and forced into the wax rim well above the incisal surface, but not so high that the baseplate will interfere with the stem being firmly imbedded in the rim.

To facilitate securing the correct relation of the lower to the upper jaw, commonly called "taking the bite," the stem should be removed and at the proper time it can again be inserted without difficulty.

Since in the use of the face bow it is necessary to locate the outer ends of the condyles in order to properly adjust the bow, and as this step requires freedom of movement of the jaw, the locations of the condyles must be found before stapling the baseplates together. The outer end of the condyle is usually located about 12 mm. anterior to, and on a horizontal plane with the external opening of the ear.

By placing the index finger at this point and instructing the patient to open and close the mouth slightly several times, any variation in location of the condyle from the point mentioned can readily be detected. Now with a blue soft pencil dot the face opposite the condyle to obviate loss of time and insure accuracy in adjusting the bow.

"Taking the bite" is usually considered a difficult undertaking, but if proper methods are employed can be uniformly accomplished with precision. The method here outlined has been followed by the writer with uniformly satisfactory results for several years. The patient should not be told of the importance of the step about to be carried out, as his voluntary assistance under such circumstances usually results in failure. He should be instructed to relax the jaw muscles so that the operator can with slight effort open and close the jaw at will. The points of the fingers of the left hand should be placed on the point of the chin, while with the right hand the lips are held apart so as to well expose the wax rims. The jaw should now be slightly depressed and closed several times. Slight pressure should be exerted upward and backward, to carry the condyles back in their sockets, but not sufficient, however, to compress the tissues in the glenoid cavity.

Under repeated opening and closing of the jaw while in a relaxed condition, the condyles will assume their normal position of rest in their sockets, and it will be noted that the lower will strike the upper baseplate in exactly the same location each time. When established, the baseplates are held in correct relation by means of two four-pointed staples, applied buccally, which when firmly imbedded in the rims prevent, not only separation, but the sliding of the plates one against the other. The relation is sometimes further secured by passing a hot instrument between the rims and slightly cementing them together in this manner. This precautionary measure is frequently resorted to, when the baseplates are large and the oral opening small, as in removal, distortion of the relation is liable to occur. When this method is followed, care should be taken to protect the lips from injury by the heated wax or spatula.

We now come to a very important and yet much neglected step, namely, that of registering the correct relation of the alveolar planes to the glenoid fossae by some means that will insure the models sustaining a similar relation to the frame hinges.

The Snow face bow fulfills the requirements mentioned in a very satisfactory manner, when properly manipulated.

The bite stem of the bow is returned to the position previously made for it in the upper wax rim fixing it solidly in place, melting the wax against it at several points if necessary. The central clamp of the bow is passed over the stem, the two condyle rods drawn out to their full extent, and the bow carried backward until the sides are horizontal. This brings the condyle rods opposite the blue dots previously marked. The rods should be pressed inward until firmly in contact with the face, and the bow shifted if necessary to one side or the other until the same number of gradations show on each rod between the face and the inner side of the bow. The clamp nuts on the rods are now tightened, and the enlarged end of each rod accurately adjusted on the pencil marks opposite each condyle. The central clamp nut is next tightened, which locks the stem firmly to the bow.

Extreme care must be exercised to prevent gravity or the torsion resulting from tightening the central clamp from disturbing the relation of the side rods with the condyles, which unless closely observed, are frequent sources of error. To overcome this difficulty the writer has designed an appliance for supporting the distal ends of the bow, which at the same time is an aid while adjusting the latter, and holds firmly, when secured, the correct relation of the several parts.

The central clamp nut being tightened, the clamp nuts in the sliding rods are loosened, the rods drawn out, the stem of the face bow attached to the baseplate firmly grasped, and the latter removed from the mouth. The sliding rods are now pressed inward to their full extent and clamped, the upper bow of the occluding frame is thrown back, and the depressed ends of the side rods placed over the hinge projections of the frame. The upper model is placed in its baseplate, the upper bow dropped down upon it, and the model attached in the usual manner. When set the entire apparatus is inverted, the lower model placed in its baseplate and attached to the lower bow in the same manner. The face bow and staples can now be removed, when the case will be ready to proceed with the registration of the condyle path.

The necessity of carrying out this step is apparent. Failure to arrange the occlusal planes of artificial teeth in the same segment or plane, or one parallell with the condyle path, will positively inhibit the normal masticatory movements, excepting the hinge motion, or if the lateral or protusive movements are employed, will result in denture displacement.

There are two possible condyle paths, one horizontal, the other inclined. The teeth are arranged in a compensating curve coincident with the lower condyle path. Now as the jaw moves forward the lower teeth are carried forward against the uppers, contact being maintained at many points, thus insuring stability of the dentures.

Suppose, however, the condyle followed the horizontal path, the arrangement of the teeth being the same as in the first instance. The result is that the lower are thrown in contact with the upper molars, while the anterior teeth are forced apart.

When the teeth are arranged on a horizontal plane and the condyle follows the upper path contact of the teeth at many points is maintained, but when the condyle takes the lower path the molars are thrown apart and the anterior teeth are brought forcibly in contact which results in a distal tipping of the dentures.

These conditions have been met and are familiar experiences with the writer in the past, and the same is no doubt true with others present. Upon being protruded, the posterior portion of the jaw drops downward on account of the inclined condyle path. This movement throws the baseplates apart distally, while the incisal borders should remain in contact the lower being protruded slightly. In practice to prevent the tipping of the baseplates, spurs of metal or rolls of wax are placed in the molar region to force each baseplate against its respective border.

Now what has occurred? The anterior surface of the condyle and the anterior margin of the lower baseplate represent two points in the periphery of the concave sphere before referred to; the condyle path and the anterior margin of the upper baseplate two points in the periphery of the convex sphere, while the forward movement of the jaw completes the example of one sphere sliding against another. By studying the diagram of the jaw in its protruded position, it will be seen that the condyle moved in a definite direction to produce a definite amount of separation of the baseplates. Any variation in amount of movement or direction would have been recorded between the baseplates by a resultant difference in space outline.

The practical steps are as follows: After mounting the models on the frame, the face bow and staples are removed from the baseplates. These are now removed from the models and on the occlusal surface of the lower wax rim a metal plate with tapering pin is inserted in the region of the first molar and well to the lingual side. This device is a suggestion of Dr. Snow to take the place of the rolls of wax.

The baseplates are now returned to the mouth and the patient instructed to protrude the jaw slightly. Three or four mm. will be ample. Too much protrusion will often result in an incorrect record of the condyle movement concerned in mastication.

Investigation shows that although the condyle path is usually curved, yet the short distance traversed by the condyle in carrying the lower teeth outward the full differential extent is so slight that practically it represents very nearly a straight line or a short cord of a large arc.

If on the other hand the condyle moves forward a considerable distance, the starting point and the terminal point of movement represent a long cord of a similar arc, comparatively speaking.

Therefore care should be taken that the condyle movement does not exceed the differential limit, or the result will be an incorrect record of the condyle path.

Pressure is now applied under the chin and the patient instructed to close the jaw, which forces the pins of the bite gauges into the upper baseplate, and brings the incisal rims of wax in contact. The staples should again be applied to insure the exact relation of the baseplates being maintained in their now partially separated condition, and the two removed from the mouth. The hub clamps controlling the condyle slots of the occluding frame should be loosened and the controlling spring thrown out of action. This permits the lower model to be moved in a spherical plane within, of course, restricted limits.

Theoretically, the upper baseplate is placed on its model, the case inverted and held in the hand, and the position of the lower model is found in its baseplates by moving the lower portion of the frame forward, thus simulating the protrusive jaw movement which occurred in taking the bite. This adjustment of the lower model to its baseplate automatically determines the slot inclination on the frame, and by holding the two models firmly in their respective baseplates, the clamps controlling the hub slots are tightened, which immovably fixes the slots in their proper position.

It will be found more convenient in practice to reverse the order of procedure here given by placing the lower baseplate on its model first, and then moving the upper model backward until it drops in position in its baseplate. The result is precisely the same.

The staples and spurs are now removed and the two baseplates separated. By throwing the spiral spring into action, the baseplates will assume their former relation to each other, the only change noticeable being a different inclination of the condyle slots which of course carries the lower model in a different and anatomically correct line of movement, a condition not possible before the registration of the condyle path was effected.

The rims of wax on both sides buccally should now be modified from flat planes to curved, and by trial with the lateral movement of the frame, made to slide against each other without contact being broken. If the spherical likeness is kept in mind the arrangement can be easily and quickly effected. The inclination lingually of the occlusal surfaces of the lower and the building down of the corresponding surfaces of the upper rims must not be lost sight of, as these surfaces properly developed assist materially in bringing the occlusal surfaces of the teeth into correct adaptation.

The steps of vital importance in this system, some of which have not been generally understood, have been briefly covered. One other still remains to be mentioned, which to some perhaps at the outset of this paper, appeared the most important of all, namely, the modification by grinding of artificial teeth.

This is a most important phase of denture construction, but regardless of how expert one ma ybe in modifying the forms of artificial teeth, if the correct foundation has not been laid, as for instance, good models, correctly mounted, on a frame that will reproduce accurately the masticatory movements, only meager success will be attained.

Little or no grinding with teeth anatomically placed, providing the teeth are of fairly good form, will produce better results for the patient than much grinding with the teeth arranged by guess-work. Combine the two, however, and results that few have dreamed of, and fewer still have realized, can be attained.

Usually the central grooves of the bicuspids and molars should be more clearly defined, not so much in an effort to deepen them as to convert the centrally inclined planes of the various cusps from rounded tubercles into somewhat broad areas of contact with the opposite teeth.

During arrangement the mesial and distal slopes of the various cusps will require modification, sometimes in their angle of inclination, and again reduction in height. Frequently the mesial or distal axial surfaces must be reduced, to correct disproportion in size between the upper and lower teeth.

The actual process of arranging the teeth in the wax is simple, and yet it must be carefully carried out. A section of wax corresponding to the width and length of a central incisor is removed, the tooth carried to place, and its labial and incisal alignment found by these surfaces of the wax rim. The wax around the pins is melted to hold it securely in place, and another section of wax is in like manner removed for the lateral or opposite central. In each case only enough wax is removed to receive a single tooth, which leaves the undisturbed rim and the tooth last placed, to serve as the alignment guide. Usually the entire upper denture is arranged first, although this is a matter of choice.

As more or less disproportion usually exists between the upper and lower teeth, it will be found more convenient for the beginner, after arranging the upper teeth as described, to place the lower second bicuspids in position first, then the first bicuspids, and on around to the centrals, then the first molars and finally the second molars.

As each tooth in the lower arch is placed in position, its occlusal form, height and transverse relation to the occluding teeth should be tested by the lateral movement of the frame. The corrections are made by grinding at such points as interfere on either the tooth itself or the opposing tooth or teeth. Special effort should be made to secure close contact of the lingual marginal ridges and cusps of the lower with those of the upper teeth on the pivotal side.

Finally the lower second molar must be so placed on the balancing side that when the jaw is moved the differential limit, its disto-buccal cusp will find contact with the mesio-lingual cusp of the upper second molar. This insures a balancing contact, indispensable for bringing out the full effective force of the muscles of mastication, and insures stability of the dentures under stress. The dominant idea is to copy nature's plan as closely as possible.

This has been called three-point contact. The term, however, while it expresses what actually exists, does not express enough, and therefore is misleading. When anatomical occlusion has been secured, this is what actually exists. On the pivotal side of the mouth, the teeth being moved to their differential position, the buccal and lingual marginal ridges of the upper and lower bicuspids are in close contact. This may be said to represent the base of a triangle extending from the first bicuspid to the second molar with contact throughout the entire extent of the base. The apex of the triangle is at the balancing point of contact. Thus the contact is grouped along the sides and apices of a three-sided figure, which no doubt gave rise to the expression "three point contact."

I am convinced that the system of anatomical occlusion of artificial teeth is now logical, practical and capable of everyday application, particularly since the last gap has been filled in such a simple and practical manner. I refer to Christiansen's method of measuring the condyle path. With each one now it is only a question of securing the proper appliances and working out the details in a practical manner, and I feel safe in saying that he who earnestly and sincerely makes an attempt along the lines mentioned, cannot fail to see the utility as well as simplicity of the method described, and will discard the plain line articulator, which is incapable of reproducing the most essential masticatory movements.—*Review*, March, 1908.

